# Electron diffraction data processing with *DIALS*

**DOI:** 10.1107/S2059798318007726

**Published:** 2018-05-30

**Authors:** Max T. B. Clabbers, Tim Gruene, James M. Parkhurst, Jan Pieter Abrahams, David G. Waterman

**Affiliations:** a Center for Cellular Imaging and NanoAnalytics (C-CINA), Biozentrum, University of Basel, Mattenstrasse 26, 4058 Basel, Switzerland; b Paul Scherrer Institute, 5232 Villigen PSI, Switzerland; c Diamond Light Source Ltd, Harwell Science and Innovation Campus, Didcot OX11 0DE, England; d STFC, Rutherford Appleton Laboratory, Didcot OX11 0FA, England; e CCP4, Research Complex at Harwell, Rutherford Appleton Laboratory, Didcot OX11 0FA, England

**Keywords:** electron microscopy, electron crystallography, protein nanocrystals, diffraction geometry, *DIALS*

## Abstract

Electron diffraction is a relatively novel alternative to X-ray crystallography for the structure determination of macromolecules from three-dimensional nanometre-sized crystals. The continuous-rotation method of data collection has been adapted for the electron microscope. However, there are important differences in geometry that must be considered for successful data integration. The wavelength of electrons in a TEM is typically around 40 times shorter than that of X-rays, implying a nearly flat Ewald sphere, and consequently low diffraction angles and a high effective sample-to-detector distance. Nevertheless, the *DIALS* software package can, with specific adaptations, successfully process continuous-rotation electron diffraction data. Pathologies encountered specifically in electron diffraction make data integration more challenging. Errors can arise from instrumentation, such as beam drift or distorted diffraction patterns from lens imperfections. The diffraction geometry brings additional challenges such as strong correlation between lattice parameters and detector distance. These issues are compounded if calibration is incomplete, leading to uncertainty in experimental geometry, such as the effective detector distance and the rotation rate or direction. Dynamic scattering, absorption, radiation damage and incomplete wedges of data are additional factors that complicate data processing. Here, recent features of *DIALS* as adapted to electron diffraction processing are shown, including diagnostics for problematic diffraction geometry refinement, refinement of a smoothly varying beam model and corrections for distorted diffraction images. These novel features, combined with the existing tools in *DIALS*, make data integration and refinement feasible for electron crystallography, even in difficult cases.

## Abstract 2

Adaptations to the *DIALS* package are described that make it a suitable choice for processing challenging continuous-rotation electron diffraction data. The results of using the extended package are presented for a case consisting of seven example data sets.

## 1. Introduction   

Electron diffraction (ED) allows the structural analysis of nanometre-sized samples of crystalline material. Since the maximal radiation dose is proportional to the sample volume, electron diffraction of organic and macromolecular compounds was long limited to two-dimensional samples (Unwin & Henderson, 1975 ▸; Hovmöller, 2017 ▸). In contrast to X-ray crystallography, the three domains, inorganic, organic and macromolecular electron crystallography, developed rather independently of each other (Vainshtein, 1964 ▸; Dorset, 1995 ▸; Kolb *et al.*, 2007 ▸; Glaeser *et al.*, 2007 ▸; Zou *et al.*, 2011 ▸). Physical and instrumental limitations, such as miniature sample size or dynamic scattering effects and lens distortions, affect data precision. However, several studies show that the model accuracy compares with that of X-ray structures (Weirich *et al.*, 1996 ▸; Mugnaioli & Kolb, 2014 ▸; Dorset, 1995 ▸; Palatinus *et al.*, 2017 ▸). Only about one and a half decades ago, electron diffraction of three-dimensional crystals was pioneered with automated diffraction tomography (ADT) and was further refined with rotation electron diffraction (RED; Kolb *et al.*, 2007 ▸; Zhang *et al.*, 2010 ▸; Gemmi *et al.*, 2015 ▸). Recently, single-crystal three-dimensional electron diffraction has also been applied to protein crystals by using the standard rotation method (Nederlof *et al.*, 2013 ▸; Hattne *et al.*, 2015 ▸; Yonekura *et al.*, 2015 ▸; Clabbers *et al.*, 2017 ▸). The only very recent use of integration software with profile fitting and scaling is indicative of the independent development of electron diffraction. These methods have been in use for decades in X-ray crystallography, improving the quality of diffraction intensities and their standard uncertainties, whilst enabling heuristic correction for systematic errors (Pflugrath, 1999 ▸; Leslie, 1999 ▸).

*DIALS* is a relatively new package for diffraction integration (Winter *et al.*, 2018 ▸) designed as an extensible toolkit for the implementation of algorithms relevant to diffraction data analysis. The core set of algorithms is presented as a suite of command-line programs that can be used following simple protocols to integrate data sets collected using the rotation method (Arndt & Wonacott, 1977 ▸). Many of these algorithms are implementations of tried and tested methods described in numerous publications over the past three decades (Bricogne, 1986*a* ▸, 1986*b* ▸; Leslie, 1999 ▸; Kabsch, 2010 ▸). However, the toolkit design of *DIALS* facilitates the construction of new algorithms (Gildea *et al.*, 2014 ▸; Parkhurst *et al.*, 2016 ▸ 2017 ▸). *DIALS* is an open-source project, allowing scientists from outside the core collaboration to contribute software or to use *DIALS* within their own projects.

To date, *DIALS* development has focused on macromolecular (MX) and chemical crystallography data sets and has been optimized for continuous-rotation data collected in fine slices using photon-counting detectors at synchrotron light sources. Despite this emphasis, with suitable modification of the parameters at certain steps, high-quality results have also been obtained for wide-sliced X-ray data sets recorded on CCD detectors (Keegan *et al.*, 2016 ▸; Khasnis *et al.*, 2016 ▸). The common fundamental assumption is that reciprocal-lattice points pass through the Ewald sphere by constant-velocity rotation around a single axis. No artificial restrictions on the diffraction geometry are imposed, allowing the modelling of diffraction experiments using a generic vectorial description (Waterman *et al.*, 2016 ▸). By default, two measurements, summation integration and three-dimensional profile fitting, are made for each reflection along with estimated errors (Winter *et al.*, 2018 ▸). The simplicity of this approach, avoiding the assumptions inherent in the details of any particular technique, means that *DIALS* is readily adapted for analysis beyond the original scope of its design.

A common feature shared between *DIALS* programs is the global modelling of an experiment, in which data are assumed to be complete before analysis begins. This has some advantages over the traditional approach of processing data by means of a moving window that passes over the complete data set in blocks of a local range of images. One is that the expensive step of integration can be performed with a high level of parallelism, as the experimental model is determined completely ahead of time. A second is that the programs can consider multiple experiments simultaneously without losing track of the connections between them. This feature has particular relevance to the global refinement of diffraction geometry, for which experiments may share some models (Waterman *et al.*, 2016 ▸), certain parameters may be constrained to shift together, or restraints may be applied between multiple crystal models. These features can be important for the analysis of electron diffraction data sets, for which determining accurate diffraction geometry may be challenging (Yun *et al.*, 2015 ▸), and current technology usually imposes the collection of incomplete wedges of data for each crystal. Here, we discuss the use of *DIALS* for the analysis of electron diffraction data that have been collected using the rotation method. As a motivational example, we describe the stages of data processing with reference to seven data sets collected at 200 keV from orthorhombic crystals of a dimeric form of hen egg-white lysozyme, as previously reported in Clabbers *et al.* (2017 ▸). These data sets are available online at https://dx.doi.org/10.5281/zenodo.1250447.

## 2. Methods and results   

### 2.1. Image formats   

The first stage in processing rotation data with *DIALS* is to import the images constituting the data set to form a DataBlock using the *dxtbx* library (Parkhurst *et al.*, 2014 ▸). This library contains format-reading classes for the majority of common file formats used in X-ray crystallography. The classes are arranged in a hierarchy from generic classes that contain code to read image data and construct an experimental model solely from metadata contained in the image headers to specific classes that may recognize a particular instrument and can override for incorrect or missing metadata. This feature is important for reading the file formats used in electron microscopy because current instruments usually do not transfer all of the information that is required to reconstruct the experimental geometry. There are three main approaches that can be taken to import electron diffraction data into *DIALS*.

(i) Externally convert the native format into a format more common for MX. This is the usual approach adopted for data processing with other programs such as *MOSFLM* (Leslie & Powell, 2007 ▸) and *XDS* (Kabsch, 2010 ▸). For example, data sets have been converted to SMV (Hattne *et al.*, 2015 ▸), PCK (Clabbers *et al.*, 2017 ▸) or CBF images (Gruene *et al.*, 2018 ▸). Where external conversion programs exist, this has the advantage that no coding or understanding of the original file format is required by the user. Often, missing metadata can be supplied during the conversion so that the resulting images contain a proper description of the experiment and no additional overrides are required when importing the data set into *DIALS*. The same set of images can then also be used with other data-processing packages. However, the reliance on an external conversion tool has some drawbacks. There is the scope for errors when metadata are introduced manually during the conversion. The proliferation of conversion tools adds complication for the user and the fidelity of the conversion process must be checked. For example, image-export functions within microscope vendor-supplied software to common formats such as TIFF might not preserve the real pixel intensities, and this fact may not be clear to the user. Even when data are properly converted, the generic readers for standard MX formats may contain assumptions that are not appropriate for electron diffraction, such as the creation of a polarized beam model. Generic readers might also not allow the desired interpretation for sophisticated cases, such as splitting a data array for a multiple-panel detector model or defining masks for certain regions of images.
(ii) Extend the *dxtbx* library to recognize native data formats. This approach entails writing a format class (typically a single, small Python module) to contribute to *dxtbx*, following the published description (Parkhurst *et al.*, 2014 ▸), and existing examples. This requires knowledge of the native data format and conventions used by *dxtbx*, as well as co­ordination with the *DIALS* developers. The advantage of investing this effort is that once included in the library, the native data format will be supported for all users with no additional conversion steps. In practice, however, where native formats lack the metadata describing the diffraction experiment, this will have to be supplied each time during data import, either by providing parameters at the command line or in a file in the PHIL format, a simple data-interchange format used within *cctbx* (Grosse-Kunstleve *et al.*, 2002 ▸). Appendix *A* [App appa] contains an example of such a file. Format classes for native file types that have now been added to *dxtbx* include image stacks in the TIA Series Data (ESD) format used by software provided with Thermo Fisher (FEI) microscopes and image stacks in Gatan DM4 format.
(iii) For local installations, testing or one-off developments for a particular data-processing problem it may be more appropriate to create a format class as a plugin rather than contributing to the *dxtbx* library. There is no difference in the procedure required to implement the class; the resulting Python module should simply be placed in a .dxtbx directory in the user’s home area and this will automatically be picked up at runtime when required. Various plugins for electron diffraction are collected at https://github.com/dials/dxtbx_ED_formats and can be downloaded and modified freely.

**Figure 1:**
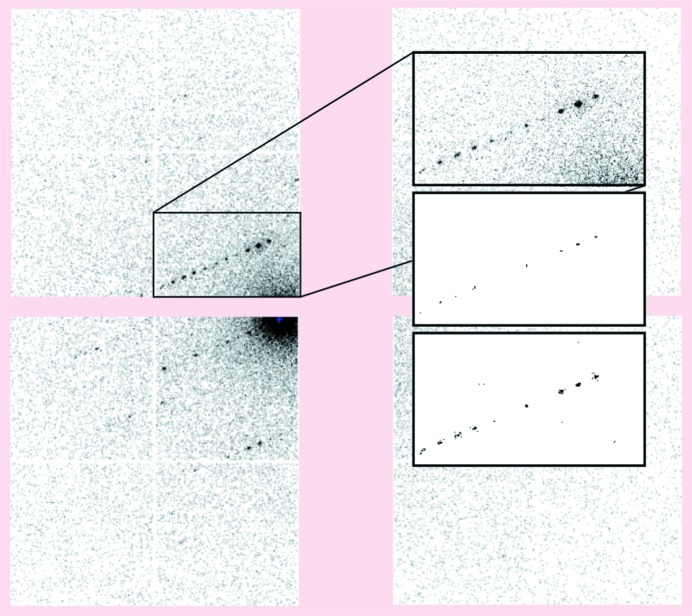
A diffraction image from data set 1 is shown using *dials.image_viewer*. The four quads have independent geometry, such that they are not forced to align on a single pixel grid. The upper inset panel shows a zoomed region of the upper left quad where a clear row of diffraction spots is visible. The middle inset panel shows the ‘threshold’ image with default spot-finding settings, which indicates which pixels will be marked as strong during the spot-finding procedure. The lower inset panel shows the same region after spot-finding settings were adjusted for this data set. In this case, this amounted to setting gain=0.833, sigma_strong=1.0 and global_threshold=1 as command-line options for the *dials.find_spots* program. The detector gain of 3.0 determined by the format class is already applied before the spot-finding operation; hence the spot-finding gain acts as a multiplier for this value.

The seven lysozyme data sets discussed here consist of diffraction images from a 1024 × 1024 pixel detector composed of a 2 × 2 array of Timepix quad detectors (Clabbers *et al.*, 2017 ▸). Large gaps between the Timepix quads are imposed by the form factor of each quad. For the original processing of these data by *XDS*, the images were converted into PCK format, in which pixel values were interpolated onto an orthogonal grid, with the gaps forming ‘dead’ areas of the image array. For processing with *DIALS* we chose a multiple-panel description instead (Parkhurst *et al.*, 2014 ▸). The images were converted to CBF (https://strucbio.biologie.uni-konstanz.de/xdswiki/index.php/Timepix2cbf) without interpretation of the gaps. We created a *dxtbx* format class specific for these images, which represents each quad as a separate panel of a composite detector. In this way, no interpolation is required because each panel has an independent position and orientation; thus, sub-pixel shifts and rotations can be represented precisely. The *dials.image_viewer* takes account of the relative position and orientation of independent panels and displays a composite image projected onto a viewing plane, as shown in Fig. 1 ▸.

A 512 × 512 pixel Timepix quad is an assembly of four abutting Timepix ASICs, each with 256 × 256 square, 55 µm pixels. However, the distance between two abutting Timepix ASICs is 350 µm, corresponding to a pitch for the abutting pixels that is about three times that of the other pixels. Since these pixels have a larger surface, they also have a higher probability of collecting more electrons. To correct for this non-uniformity, the conversion to CBF splits pixels with an *x* (and/or *y*) coordinate that equals 256 or 257 into three pixels that are 55 µm wide (or high). This results in 516 × 516 pixel frames with a discernible, six-pixel wide cross, in which the pixels have a gain that is about three times higher than that of the other pixels outside the cross. This was corrected by multiplying the counts of the unaffected pixels by a factor of three. As the Timepix detector is operated in electron-counting mode, the converted images therefore model a detector with Poisson response and a multiplicative gain of 3.0. This was recorded in the *dxtbx* format class so that the correct gain value would be used automatically, for example in the calculation of error estimates for integrated intensities.

### 2.2. Spot finding   

The spot-finding algorithm used in *DIALS* is rather sensitive to the detector gain. No automatic evaluation of the gain is performed prior to spot finding, although a value can be determined using the program *dials.estimate_gain*. This uses the mean and variance of pixels within a region of interest (Leslie, 2006 ▸) and may significantly underestimate the true gain for detectors that have a non-negligible point spread or corrections applied that reapportion signal between neighbouring pixels (Waterman & Evans, 2010 ▸). If the correct gain is known it is usual for this to be set by the format class used to import images. Otherwise, a suitable value should be passed to *dials.find_spots* for use by the spot-finding algorithm. In difficult cases it may be necessary to optimize the gain and other spot-finding parameters, the effects of which can be explored interactively using *dials.image_viewer*. For the seven example data sets discussed here we typically found that it was necessary to increase the sensitivity of spot finding and then reduce additional noise by using a global threshold. Appropriate spot-finding settings were determined manually for each data set separately. The effect of these settings for data set 1 is shown in Fig. 1 ▸.

### 2.3. Experiment geometry   

The most substantial difference between the processing of rotation data from electron diffraction compared with X-ray diffraction lies in the modelling of the diffraction geometry. The short wavelength of an electron beam (0.02508 Å for 200 keV electrons compared with 1.0332 Å for 12 keV X-rays) implies a correspondingly large Ewald sphere, with a small 2θ scattering angle even for the highest resolution reflections.

The low diffraction angles imply that a large effective sample-to-detector distance is needed to magnify the diffraction pattern and achieve sufficient spatial separation between peaks. Large detectors are advantageous for crystallography because they allow the sample-to-detector distance to be increased, which both reduces diffuse background and improves the spatial separation of the peaks (Stanton, 1993 ▸). However, the detector distance is limited in a transmission electron microscope (TEM) by the largest possible magnification and the relatively small size of the detectors. Whilst the true camera position underneath the TEM column is always at a fixed distance, the effective detector distance is set by the projector lens system and does not correspond directly to a quantity that can be measured mechanically. Similar to an X-ray beamline, the sample-to-detector distance in a TEM is easily calibrated with reliable test crystals. However, inaccuracy in the recorded effective distance may be difficult to correct by the usual process of diffraction geometry refinement owing to the high correlation between unit-cell parameters and the detector distance when 2θ_max_ is small, in which case the Ewald sphere is almost invariant with respect to linear scale (see §[Sec sec2.6]2.6; Van Genderen *et al.*, 2016 ▸). In addition, imperfections in the lens system may introduce distortions in the recorded diffraction images. By disregarding such defects, which are discussed further in §[Sec sec2.4]2.4, the processing software ignores the lens system and models the experiment with an effective detector distance.

**Figure 2:**
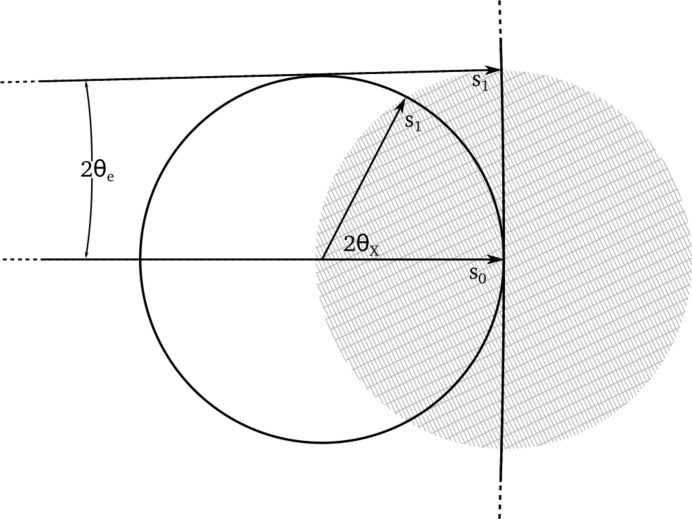
The Ewald constructions for the electron diffraction and X-ray cases are compared. The cross-hatched circle represents a reciprocal lattice within a limiting sphere of 1 Å resolution. The Ewald sphere for 12 keV X-rays with a wavelength of 1.0332 Å is represented as a complete circle, with the scattering vector **s**_1_ drawn at the 1 Å limit, forming an angle of 2θ_X_ = 62.2° from the incident beam direction along **s**_0_. At this scale, the Ewald sphere for 200 keV electrons, with a wavelength of 0.02508 Å, cannot be shown as a complete circle as it has a radius over 40 times greater. The equivalent scattering vector **s**_1_ for 1 Å diffraction forms an angle of only 2θ_e_ = 1.44° from the incident beam direction. It is worth noting that the reciprocal lattice is sampled along an almost planar surface, implying that data from a single image contain no information about the reciprocal-lattice dimension in the direction along the incident beam.

The relatively extreme geometry of electron diffraction is unfamiliar to many X-ray crystallographers. It is instructive to compare graphical schematics, such as Fig. 6 in Clabbers & Abrahams (2018 ▸) for the real-space geometry of the instruments and Fig. 2 ▸ here, for a comparison of the Ewald construction in reciprocal space for the two cases.

Another potential source of inaccuracy in the initial model for the diffraction geometry arises because of the relatively poor characteristics of the sample-positioning stage of electron microscopes compared with X-ray goniometers for the purpose of rotation-method experiments. Improved setups are possible, but are not widely available (Yonekura *et al.*, 2015 ▸; Shi *et al.*, 2016 ▸). The rotation range per image is generally assumed to be constant and accurate. Instruments used for electron diffraction should therefore be well calibrated (Gemmi *et al.*, 2015 ▸). Small, smooth deviations from the expected rotation angle can then be modelled in *DIALS* as part of the scan-varying refinement of the crystal.

**Figure 3:**
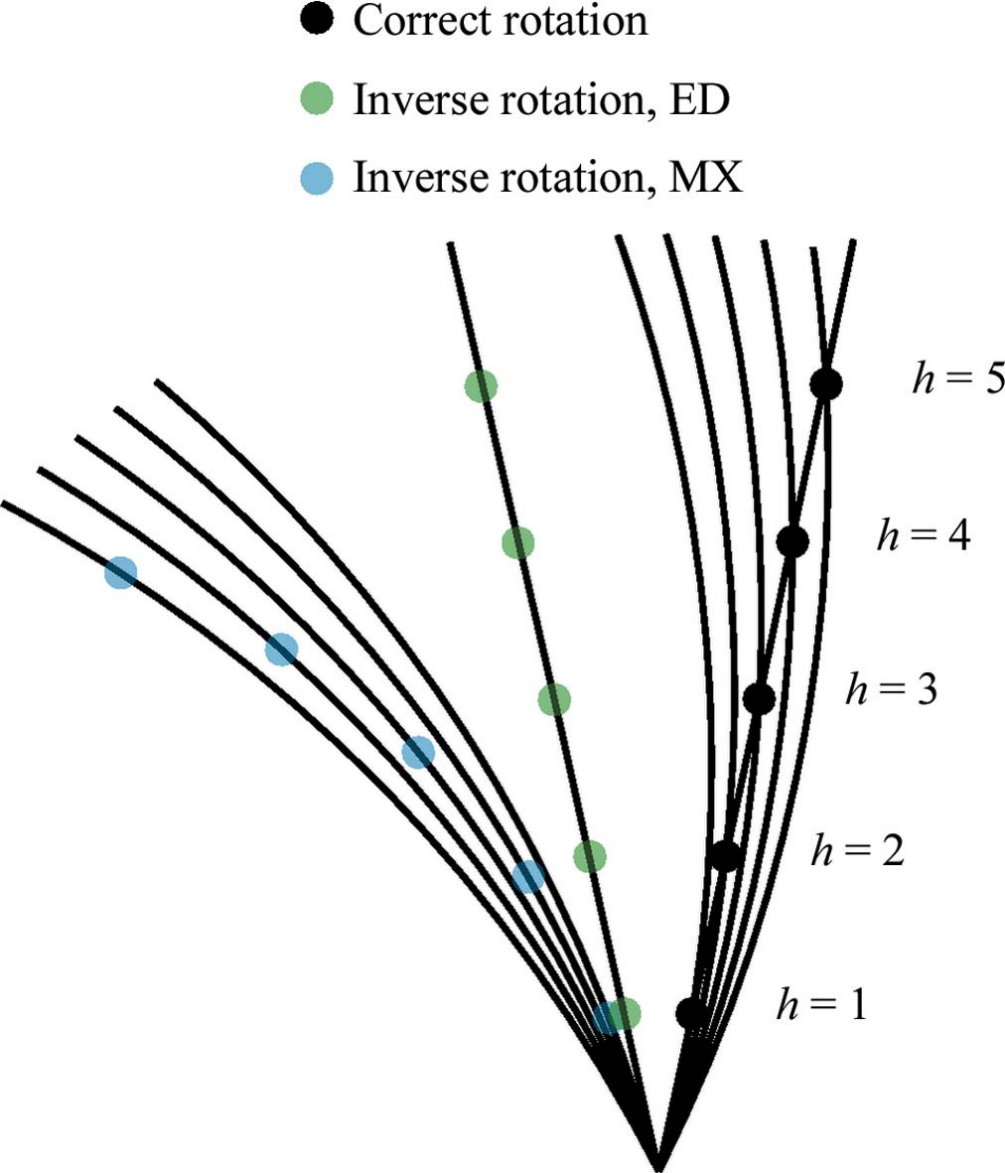
Five reciprocal-lattice points are shown (in black and labelled) along the *a* ^∗^ axis for a crystal with unit-cell dimension *a* = 10 Å. Arcs representing the surface of the Ewald sphere with a typical X-ray wavelength of λ = 1.0332 Å intersect these points at rotation angles between 15.0° for *h* = 1 and 27.0° for *h* = 5, where rotations are assumed to be clockwise from vertical in the plane of the figure. If the modelled rotation axis is inverted then φ centroids of observed spots would be mapped onto Ewald spheres rotated between −15.0 and −27.0°, resulting in a distinct curvature to the reconstructed reciprocal lattice (points shown in blue). In the case of electron diffraction at λ = 0.02508 Å the spots are observed almost simultaneously at rotation angles between 12.1 and 12.4°. For clarity a single Ewald arc is shown for *h* = 3. If the assumed axis is inverted then φ centroids between −12.1 and −12.4° still result in almost a straight line (points shown in green). It is therefore difficult to determine the correct direction of rotation from the appearance of the reconstructed reciprocal lattice alone.

Generally, there may be uncertainty regarding the orientation of the rotation axis, the direction of rotation and the rotation range per image. Procedures have been developed to identify rotation-axis orientation for electron diffraction studies (Dorset, 1976 ▸; Kolb *et al.*, 2009 ▸); however, there is no implementation of an automated algorithm for this in *DIALS*. Nevertheless, for macromolecular samples there are a relatively large number of spots found throughout a data set and these can be used to obtain a reasonable estimate of the rotation-axis orientation in the plane of the detector by inspecting the images. This axis forms a line through the beam centre along which reflections have the widest reflecting range, and few reflections are found. As long as the initial estimate is good enough for successful indexing, the remaining error may be corrected by the geometry refinement procedure described in §[Sec sec2.6]2.6. The direction of rotation around the axis is more difficult to determine. For an X-ray experiment the curvature of the Ewald sphere makes the incorrect choice obvious, for example using a visual tool such as *dials.reciprocal_lattice_viewer* (Winter *et al.*, 2018 ▸). By contrast, the flatness of the Ewald sphere in electron diffraction ensures that either choice of handedness of rotation will produce regular reciprocal-lattice positions, as shown in Fig. 3 ▸. If indexing is successful, it is likely to work either way. For any case where there is ambiguity, the inverse direction should also be tested and the results compared. The correct solution will have a lower r.m.s.d. for the angular residual between the predicted and observed positions of the reflections.

### 2.4. Image distortion owing to lens effects   

Image distortion is not unique to electron crystallography. In X-ray crystallography, geometrical distortions may be present owing to components of the detector system. A familiar example of these are spatial distortions introduced by the fibre-optic taper in a phosphor-taper CCD area detector (Stanton *et al.*, 1992 ▸). In this case, the distortion is a fixed property of the detector and it is usual for images to be corrected by manufacturer-supplied routines prior to analysis. Nevertheless, data-processing packages such as *XDS* have facilities for applying a distortion correction in the form of look-up tables. Even with the advent of hybrid pixel-array detectors, which have a direct coupling between the detector surface and the counting electronics, geometrical distortion may be used to correct for subpixel shifts and misorientations between the modules of the detector array. In electron crystallography, geometrical distortions of the detector are no less relevant, while there is the additional factor of the possibility of distortion of the diffraction pattern itself owing to effects of the electron optical system. Possible distortions include anisotropic magnification, where the diffraction pattern is elongated in one direction, transforming a circular powder pattern into an ellipse (Capitani *et al.*, 2006 ▸; Clabbers *et al.*, 2017 ▸). Care must be taken to investigate the presence of these effects in electron diffraction data sets and, as they are not mechanical properties of the instrument, it is necessary to recalibrate when instrument settings are changed.

Despite the fact that the distortion occurs in the direction of the scattered rays rather than as a property of the detector, it is reasonable to correct images using the same means as for other sources of distortion. Within *DIALS*, we implemented a similar mode for distortion correction as used in *XDS*. A pair of distortion maps encode the pixel offset across the detector for both the fast and slow directions. These maps are equal in size to the pixel array of the detector (for a multiple-panel detector the correction files encode a list of separate maps for each panel). No interpolation is performed during the application of the distortion maps. In principle, sharp changes to correct for shear defects would be possible; however, for the case of lens abberation the offset varies slowly over the face of the detector so that neighbouring values in the look-up table are similar. The distortion maps are applied during the conversion between detector pixel coordinates and virtual detector millimetre coordinates. During the transformation from millimetre coordinates to pixel coordinates, the uncorrected pixel coordinate is first calculated and the correction is applied to obtain the distortion-corrected pixel coordinate. Likewise, during the transformation from pixel coordinates to millimetre coordinates the reverse correction is first applied and the millimetre coordinate is calculated from the reverse corrected pixel coordinate.

Data sets 2–7 in our examples all showed a significant elliptical distortion, which was constant across these data sets. The parameters of this distortion were determined using a well known diffraction standard, as described previously (Clabbers *et al.*, 2017 ▸). The use of an independent standard for calibration is good practice that would become essential in the case where the sample of interest has an unknown unit cell. We extended the program *dials.generate_distortion_maps* to produce *X* and *Y* distortion maps for the four-panel detector model based on the known parameters. These maps were registered for each relevant data set during the *dials.import* step, after which they were loaded and applied automatically whenever required by *DIALS* programs.

### 2.5. Indexing   

Provided that a sufficient number of strong spots have been collected (*cf.* §[Sec sec2.2]2.2), indexing of electron diffraction works with similar reliability as for X-ray diffraction data. Difficulties mostly arise from systematic errors such as the stability of the rotation axis and the often large variation in the oscillation width Δφ. The default method for determining the unit-cell basis vectors in the *dials.index* program is based on the three-dimensional FFT of found spot positions, which works well even when the scan consists of a relatively narrow wedge, as is typical for an electron diffraction data set. The program *dials.index* performs refinement of the initial solution; therefore the guidance listed in §[Sec sec2.6]2.6 for refinement of ED geometry is also relevant and it is possible to pass options for the *dials.refine* program into *dials.index* where required.

Unless a model space group was chosen by the user, the indexing results are presented with triclinic symmetry. The compatibility of other choices of Bravais lattice with the triclinic solution can be tested using the program *dials.refine_bravais_lattice* (Winter *et al.*, 2018 ▸; Sauter *et al.*, 2006 ▸). There is no difference in usage compared with X-ray data; however, for electron diffraction the results might be more difficult to interpret. In particular, the metric fit reported for each trial solution (Le Page, 1982 ▸) may be large (for example greater than 1°) even for a correct solution, whereas much smaller values are expected for good-quality X-ray data. The correlation coefficients between intensities related by symmetry operations of the lattice are affected by low multiplicity of the data and by factors that cause deviation from expected intensities such as dynamic diffraction. As a result, these are not as useful in deciding on the correct lattice as they are in X-ray experiments. The key criterion is then the r.m.s.d. between predictions and observations. A pool of solutions with r.m.s.d.s similar to the original triclinic solution are good candidates. Any solution resulting in a significant increase in r.m.s.d. is suggestive of an over-constrained lattice and should be discarded.

For six of the seven example data sets, indexing followed by the selection of an orthorhombic lattice was successful with default options apart from fixing some detector parameters, as described in §[Sec sec2.6]2.6. For data set 6 we additionally fixed the beam orientation parameters and provided the expected unit cell and a restraint to this target cell during refinement. This data set shows relatively poor diffraction. Rather few spots were successfully indexed and r.m.s.d.s between the predicted and observed rotation angles remained high after refinement (see Table 1 ▸). The action of both constraints and restraints help to stabilize and guide refinement in such difficult cases.

### 2.6. Global refinement of the unit-cell and instrument parameters   

Following indexing, the model for the diffraction experiment geometry is further refined. This consists of the joint refinement of global parameters, including the beam direction, the unit-cell parameters, the cell orientation and the detector position and orientation. The choice of refined parameters is left to the user, with the default set being appropriate for typical X-ray data sets. For details, see Waterman *et al.* (2016 ▸). The flexible geometry description and refinement procedures of software such as *XDS* or *DIALS* is of great importance in electron diffraction studies, where the initial geometry may be quite poor. The radius of convergence of these procedures is high enough to correct large errors, as long as the indexing of spots is correct. In common with X-ray data processing with *DIALS*, it is usual to first refine a ‘static’ model for the whole data set, in which parameters such as the crystal unit cell and orientation angles are not allowed to vary across the scan. The global refinement of a data set improves the stability of the refinement procedure. However, the geometry of an electron diffraction experiment raises particular issues that should be taken into account, especially if the data quality is limited by low-resolution diffraction for some or all of the scan or poor-quality spot centroids, or if the scan is an especially narrow wedge. In this section, we offer some practical advice for *DIALS* refinement tasks with challenging electron diffraction data.

It is more difficult to refine unit-cell parameters using electron diffraction data than using X-ray data. This is mainly caused by the weaker signal and the much smaller diffraction angles 2θ_max_ in electron diffraction.^1^ A weak diffraction signal implies fewer diffraction spots and lower accuracies in determining their centroids, compromising the accuracy of the refinement. The small diffraction angle implies a low Ewald sphere curvature and a very high correlation between detector distance and a uniform unit-cell scale factor. In the limiting case the relative accuracy of the unit cell scales linearly with the relative accuracy of the detector-distance calibration. In cases where unit-cell imprecision does not prevent structure solution, the parameters can be adjusted during model refinement (Gruene *et al.*, 2018 ▸). Automatic options for performing this have recently been implemented in *REFMAC*5 (see §[Sec sec2.10]2.10).

The high level of correlation between parameters in diffraction geometry refinement problems has long been recognized. The method of eigenvalue filtering was proposed to allow refinement to proceed in such cases (Reeke, 1984 ▸; Bricogne, 1986*b* ▸) by automatically selecting only those parameters, or linear combinations of parameters, that have the greatest effect at each step of refinement. This was deemed to be necessary at the time to refine crystal parameters using data from a single oscillation film. Within *DIALS*, all available data are used for a global refinement. This reduces correlations and provides a better determination for parameters when the scan range is wide; thus, the default behaviour is to refine the beam, crystal and detector parameters simultaneously, which works well for X-ray data. We have seen that when limited to a narrow wedge of data recorded with the geometry of the electron diffraction experiment, high correlations are again problematic. *DIALS* refinement does not use the eigenvalue-filtering method, but by default uses a Levenberg–Marquardt algorithm, which provides an alternative approach for dealing with near-singular least-squares problems. In practice, we find that this algorithm is robust even in the presence of very high parameter correlations. However, experience shows that the most challenging problems with electron diffraction geometry may need many steps before convergence is achieved, where this is defined as a negligible further reduction in r.m.s.d.s. For this reason, from *DIALS* v.1.8 the maximum number of iterations before refinement terminates has been raised to 100 from 20 for the Levenberg–Marquardt algorithm (the limit can always be adjusted by the user *via* the max_iterations parameter).

If a good estimate for the unit cell is available as prior knowledge, this can be incorporated into refinement by the use of restraints, tying the unit-cell model to an external target. Unit-cell restraints are currently available for static refinement of unit-cell models but not scan-varying refinement, as they were originally developed for XFEL serial crystallography where scan-varying refinement is irrelevant. The unit-cell parameterization in *DIALS* is expressed with reciprocal metrical matrix elements as parameters (Waterman *et al.*, 2016 ▸). However, for ease of use, restraints are specified in terms of the real-space cell, as shown by the example given in Appendix *A* [App appa]. Each crystal included in refinement can add up to six restraint terms (for the triclinic case). Irrelevant restraints for unit-cell parameters that are already constrained by lattice symmetry are automatically excluded. Every restraint term adds a pseudo-observation to refinement. Taking the unit-cell parameter *a* as an example, the pseudo-observation term *R*_*a*_ consists of the squared residual between this parameter and its target value *a*_t_ with a weighting factor. In common with the real observations, the first derivatives of the pseudo-observations with respect to the refinable parameters (here arbitrarily denoted *p*) are also required for refinement by nonlinear least-squares methods,
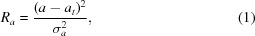


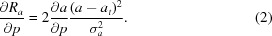



In principle, statistical weighting could be achieved by setting the weights equal to the inverse variance of the target unit-cell parameter values. However, numerical uncertainties from refinement are known to be underestimated (Dauter & Wlodawer, 2015 ▸). For X-ray diffraction refinement we usually try values between σ ≃ 0.001 for qualitatively ‘strong’ restraints and σ ≃ 0.1 for ‘weak’ restraints, monitoring the effect on the refined r.m.s.d.s. In the electron diffraction case setting even very weak restraints to a target cell can avoid issues with the unit cell and detector distance drifting when these are refined simultaneously. Nevertheless, the high correlation between these parameters means that the problem of distinguishing between cell volume and detector distance remains salient, and indeed the unit cell can be driven towards a target cell of incorrect volume with a minimal increase in refined r.m.s.d.s if the detector distance is also refined. It is generally advisable to accurately calibrate the effective detector distance prior to ED data collection and then to fix this during data processing. Other parameters that it may be prudent to fix include the detector τ_2_ and τ_3_ values, which describe rotations around axes in the plane of the detector, similar to *MOSFLM*’s TILT and TWIST. Joint refinement of these parameters along with the beam direction and detector translations within the detector plane can be unstable.

For six of the example data sets, fixing the detector distance, τ_2_ and τ_3_ gave acceptable results for joint refinement of the beam, crystal and detector in-plane translation and rotation parameters. For the more difficult case, data set 6, no additional parameters were fixed, but a restraint to the target cell as given in Appendix *A* [App appa] was used. Only 139 reflections were available for refinement in this case after outlier rejection. The use of the restraint ensured that the refined cell remained reasonable. In particular, without the restraint the long axis dimension drifted to above 108 Å. Including the restraint increased the r.m.s.d.s in *X* and *Y* by less than 0.07 and 0.14 pixels, respectively, and had a negligible effect on the r.m.s.d. in the rotation angle, demonstrating a case in which this feature can be used to guide refinement without resulting in a model that stands in dispute with the centroid data.

### 2.7. Scan-varying refinement of crystal and beam parameters   

In a typical use of *DIALS*, the global static model for a data set is used as a starting point for scan-varying refinement. As originally implemented (Waterman *et al.*, 2016 ▸), this was intended to capture changes to the crystal unit-cell and orientation parameters during data collection. These parameters were allowed to vary in a smooth manner by evenly distributing sample points across the scan and interpolating values at any one position using a Gaussian smoother. The beam and detector parameters could be jointly refined to global, static values alongside the scan-varying crystal.

The analysis of electron diffraction images raises a new issue in that instrument stability during the course of data collection cannot be simply assumed, as it is for MX data. In some cases, there is significant drift of the beam centre during data collection caused by instability of the alignment or charging effects. Previous methods to handle this involve procedures to identify the shift for each image and write out corrected images in which the beam centre remains constant, effectively describing the drift in terms of shifts of the detector (Wan *et al.*, 2013 ▸; Nederlof *et al.*, 2013 ▸; Hattne *et al.*, 2015 ▸). The procedures differ in the way that the beam centre is determined for each image. In the simplest case, the high scattering cross-section for electrons allows, for some instrumentation, the direct beam to be recorded simultaneously with diffraction spots, avoiding the need for a beam stop. When images are not corrected, software such as *MOSFLM* or *XDS* can be set to independently refine the beam centre for each image or within small blocks of images. The focus on global refinement in *DIALS* means that an alternative approach was sought. Beam drift in electron diffraction experiments, at least those collected by a continuous rotation protocol, appears to occur gradually. Therefore, it seems reasonable to assume that a smoothly varying model for the beam-direction vector would suffice to represent this effect. For small magnitudes of the total drift, the difference between correction by implicit detector shifts and modelling of a drifting beam will be negligible. For the purposes of ED data processing, we extended the scan-varying refinement methodology from crystal parameters to optionally also apply to the beam parameters; this is available from *DIALS* v.1.9 onwards.

The difficulties with refinement inherent to electron diffraction geometry are exacerbated during scan-varying refinement. Like static refinement, scan-varying refinement in *DIALS* is also global, in that data from the full rotation scan are used in a single optimization procedure. However, at any point in the scan the local values for the crystal unit cell, angular misset and potentially the beam direction parameters are dominated by the data close to that point. Spot centroids at rotation angles further from that point have a diminishing effect on the local model, controlled by a Gaussian smoother. While this allows the model to express genuine smooth changes, it reduces the stability of the refinement procedure. This has been seen in cases where a static crystal model allows global refinement of both the detector and crystal parameters to reasonable values, but scan-varying refinement of the crystal results in a drift of the average unit-cell volume and detector distance. Despite these observations, scan-varying refinement is still preferable to static refinement of the beam, crystal and detector models within local narrow wedges, which suffers even more from high parameter correlations. To stabilize a problematic scan-varying refinement task we must either restrain or constrain (fix) some parameters of the model. There is no automatic determination of a suitable parameterization for refinement in *dials.refine*. Diagnostics (see §[Sec sec2.8]2.8) may help to understand the details of a particular case and guide choices; however, ultimately the user must inspect the resulting models for reasonable geometry as well as the final r.m.s.d. values.

We performed scan-varying refinement prior to integration for the seven example data sets. A variety of protocols was tested, and the best was chosen for each data set according to merging statistics after scaling of that data set in isolation by *AIMLESS* (Evans & Murshudov, 2013 ▸). In each case, we fixed all detector parameters so that the detector maintained the geometry from the static refinement step. For data set 1 a significant drift of the beam centre was observed. We enabled scan-varying refinement of both beam direction angles μ_1_ and μ_2_ in the nomenclature of Waterman *et al.* (2016 ▸). Remarkably, the simplest model consisting of two refineable sub­parameters for each angle resulted in the best merged data set, rather than models with more subparameters that are smoothed less in order to track higher frequency changes to the beam drift. Scan-varying refinement of the beam was tested for each of the other data sets. For two cases, data set 4 and data set 5, merging statistics favoured static refinement of the beam direction. In the other cases, the simple two-subparameter model for each beam angle was used. For each data set, the three crystal orientation ‘misset’ angles were refined in a scan-varying manner, using default smoother parameters. A scan-varying unit cell was refined for each case, except for data sets 3, 4 and 6, for which refining a global, static cell stabilized refinement and produced better merging statistics. Further details of the diffraction geometry modelling for each data set are given in Table 1 ▸.

### 2.8. Diagnostics for problematic diffraction geometry refinement   

§§[Sec sec2.6]2.6 and [Sec sec2.7]2.7 describe parameters that need to be adjusted in difficult cases. To date, even electron diffraction data sets from standard proteins may be found to be difficult (Clabbers *et al.*, 2017 ▸; Hattne *et al.*, 2015 ▸). At this early development stage, diagnostic tools are important for fine-tuning parameters. The program *dials.refine* provides some facilities for investigating the main issue that we have identified, namely the high level of correlation between the effects of different parameters on the model. This information is contained within the Jacobian matrix built up as part of each step taken by the nonlinear least-squares optimization algorithm. In this section, we present two diagnostics based on analysis of the Jacobian matrix and pick out the salient differences that occur simply as a feature of the refinement of geometry at the very short wavelength typical for electron diffraction.

Each step of the nonlinear least-squares problem is expressed as a linearized subproblem of the form
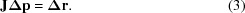



By convention, the three-dimensional observations are split so that **Δr**, the vector of residuals, contains first the (*X* − *X*_o_) components, followed by the (*Y* − *Y*_o_) components and finally the (φ − φ_o_) values. **J**, the Jacobian matrix of first partial derivatives of the residuals with respect to each parameter of the problem, is thus similarly formed in blocks, with the upper third of the matrix corresponding to ∂*X*/∂*p* values, the second to ∂*Y*/∂*p* and the lower third to ∂φ/∂*p*. The vector **Δp** is the parameter shift vector to be determined for the step.

The first diagnostic consists of graphical ‘corrgrams’, which are a way of rapidly assessing correlations between the parameters of refinement in a visual manner. The data represented by a corrgram consist of the matrix of pairwise correlation values calculated between columns of the Jacobian. Since its introduction, described in Waterman *et al.* (2016 ▸), this diagnostic has been improved. Rather than calculating a single corrgram using correlation between each full column of the Jacobian, the three-dimensional nature of the centroid data is respected and three corrgrams are produced: one for each of the blocks of the Jacobian, corresponding to the dimensions *X*, *Y* and φ. These separate figures are more appropriate for assessing the levels of correlations between parameters implied by the data, whereas a single corrgram can obscure these features. This is because the derivatives of calculated centroid positions with respect to some parameter ∂*X*/∂*p*, ∂*Y*/∂*p* and ∂φ/∂*p* come from different distributions and thus should not be combined in a meaningful calculation of correlation.

While the corrgram diagnostic qualitatively identifies which parameters are the least distinguishable from each other, it might still not give a clear indication of which refinement cases will actually cause problems. Certain correlations are high anyway even in unproblematic cases. For this reason we also investigated an alternative, quantitative, diagnostic with a simpler interpretation, namely the condition number of the Jacobian matrix **J**. This provides a measure of how well posed the subproblem given by (3) is, but does not pick out which parameters are culpable. A condition number κ(**J**) of infinity means that **J** is singular, while a finite value of κ(**J**) gives a bound on the accuracy of the solution to (3).

The Jacobian used to calculate both the corrgram and the condition-number diagnostics does not include any additional blocks related to pseudo-observations that may be used as restraints in refinement. For this reason, it should be noted that the diagnostics give information about the underlying degeneracy of parameters determined only by the geometry of the problem, not including the effects of modifications to the problem that may have been introduced to improve the robustness of the procedure. Similarly, the diagnostics inform us directly about properties of the normal equations of the Gauss–Newton problem implied by (3) rather than the modified normal equations of the Levenberg–Marquardt algorithm that is typically in fact used to find the solution. This ensures that these diagnostics can be used to warn us of problems with the setup of the diffraction geometry refinement itself, without conflation with factors relating to implementation details of the algorithm used to perform the optimization.

To investigate the difficulties faced with refinement problems that are solely a result of the electron diffraction geometry, we elected to perform refinement against simulated data. In this way, we could compare two refinement procedures using an identical crystal model, beam direction and rotation axis, while altering the wavelength and detector distance to match typical values for electron diffraction in one case and X-ray diffraction in the other. Details of how the simulated data were constructed are presented in the Supporting Information. Refinement was performed for the same sets of reflections with both versions of the geometry, using default settings in *dials.refine*. In each case 13 parameters were refined in total: six to describe the detector position and orientation, one beam orientation angle, three crystal orientation angles and three reciprocal metrical matrix elements for the unit cell. For the final step of refinement prior to termination at r.m.s.d. convergence, corrgrams were produced and the condition number calculated for comparisons.

**Figure 4:**
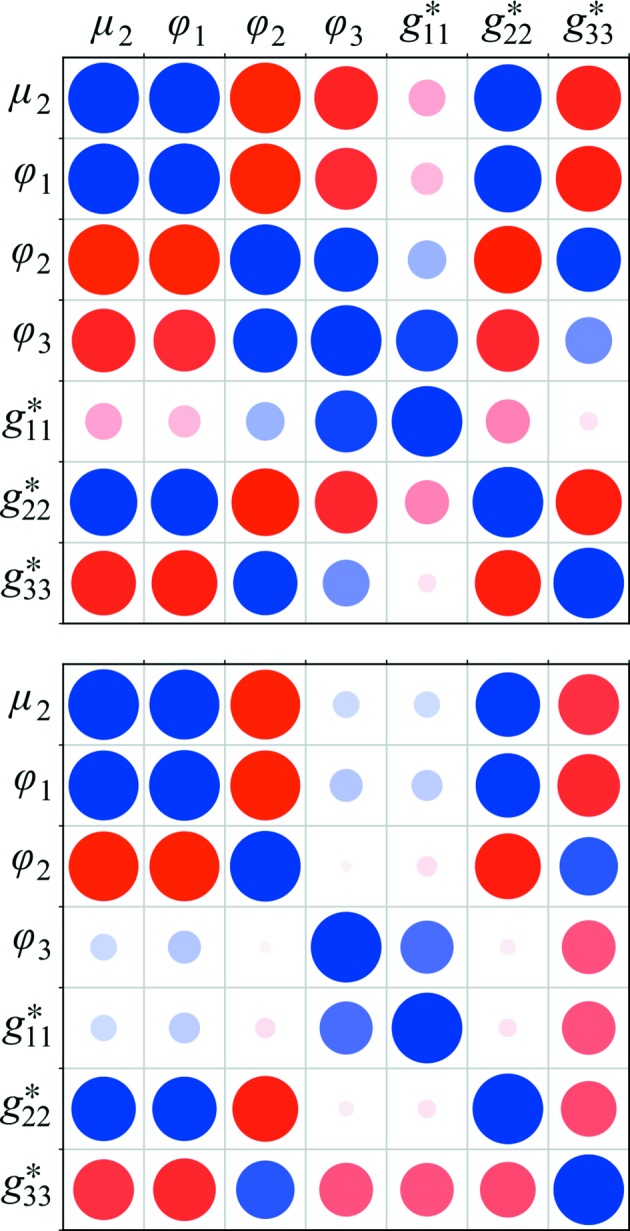
Geometry refinement against simulated data was performed assuming either typical electron diffraction geometry or X-ray diffraction geometry, as described in the text. Corrgrams were produced for the final step of refinement to provide immediate visual feedback regarding correlations between the effects of refined parameters on the model. The colours and areas of the circles are related to the values of the correlation coefficient, with large blue circles indicating strong correlation and large red circles indicating strong anticorrelation. This plot shows the correlation between the effects of different parameters on the angular residuals (φ − φ_o_), with the refined detector parameters excluded from the plots as they have no effect on the φ residuals. The parameter labels are as defined in Waterman *et al.* (2016 ▸). The upper panel shows the corrgram for the electron diffraction geometry and the lower panel shows the equivalent corrgram for the X-ray diffraction geometry.

The complete two sets of three corrgrams are shown in Supplementary Fig. S1. The pattern of high correlations between parameters that affect the predicted reflection positions (*X*, *Y*) on the detector plane are similar in the cases of electron and X-ray diffraction geometries. However, in general, the absolute values of correlations are higher for the electron diffraction geometry. The most striking difference between the two cases is shown on the corrgram for the parameters that affect the predicted rotation angle φ_c_. None of the detector parameters affect φ_c_, so only the beam and crystal parameters are of interest. The relevant subset of the corrgram is reproduced in Fig. 4 ▸. This figure shows that absolute correlations between certain parameters are high in either case, but that the electron diffraction geometry shows increased absolute correlations between φ_3_, the crystal orientation around the *Z* axis, and other parameters. In general, absolute correlations are smallest between the parameter *g* ^∗^_11_, here corresponding to the short axis of the cell, and other parameters for either version of the geometry. For this data set, the short cell axis was aligned closest to the rotation axis. As a result, this dimension is relatively well determined by centroid data from images throughout the data set. However, even for this parameter the electron diffraction geometry produces larger absolute correlations with other parameters, except one, *g* ^∗^_33_, which parameterizes the long axis of the cell. Detailed interpretation of these plots is difficult and requires complete knowledge of the definitions of each of the parameters, including the directions about which they are defined and the order in which they act to compose the final model. Broadly, however, we can immediately see a pattern of greater magnitude correlations for the electron diffraction case and would expect a correspondingly more challenging refinement problem.

The second diagnostic provides a measure to quantify this effect. The condition number at the final step of refinement for the electron diffraction geometry κ(**J**_ED_) ≃ 8 × 10^5^, while for the X-ray diffraction geometry κ(**J**_MX_) ≃ 2 × 10^3^. This clearly indicates that the electron diffraction geometry presents a considerably less well posed problem for refinement. With the simulated data we jointly refined 13 parameters simultaneously; however, for the processing of the seven real example data sets we fixed the detector distance, τ_2_ and τ_3_ parameters to stabilize refinement and avoid the cell volume drifting away from reasonable values. The condition number quantifies this stabilization. When the same parameters are fixed during refinement of the electron diffraction geometry against simulated data this reduces to κ(**J**_ED_) ≃ 8 × 10^3^, a two order-of-magnitude improvement of the problem condition.

The diagnostics presented here can help to design protocols for successful diffraction geometry modelling in difficult cases. However, there is much variation between data sets, and not yet enough experience to allow generalization or automated selection of an optimal protocol. For the example data sets, the best procedure we found was to rely on careful, independent calibration of distance and fix that during refinement. Other parameters were additionally fixed for individual cases. Small errors in the cell can be tolerated for the purposes of integration and improved later at the stage of model refinement.

### 2.9. Integration and data reduction   

Following global modelling of an experiment using *dials.refine*, data are integrated with *dials.integrate*. No special options are required for integrating electron diffraction data. For the examples we specified options only to run multiple processes in parallel and to specify resolution limits within which all spots will be predicted for integration. The integrated data sets were then exported to MTZ format using *dials.export* and combined by *POINTLESS* (Evans, 2006 ▸) for scaling and merging together with *AIMLESS* (Evans & Murshudov, 2013 ▸). This procedure included a reindexing step to convert the orthorhombic cell used for integration, with *a* < *b* < *c*, to the conventional space group *P*2_1_2_1_2. Merging statistics are summarized in Table 2 ▸, while Supplementary Table S1 summarizes statistics for data sets scaled individually.

### 2.10. Structure solution and refinement   

The structure was determined as described in Clabbers *et al.* (2017 ▸), with the exception of using the intensities for molecular replacement in *Phaser* (McCoy *et al.*, 2007 ▸; Read & McCoy, 2016 ▸), and with the additional step of refining the lattice in *REFMAC*5 (Murshudov *et al.*, 2011). Lattice refinement allows the unit cell to be refined by a single scaling factor independent of the sample-to-detector distance, thus removing the ambiguity between detector distance and lattice parameters. The newly found unit cell was then used for subsequent structure refinement and validation (Murshudov *et al.*, 2011 ▸; Joosten *et al.*, 2014 ▸; Luebben & Gruene, 2015 ▸).

## 3. Discussion and conclusions   

Electron diffraction from three-dimensional crystals has recently been used to solve the structures of macromolecules such as proteins. Previous authors have shown that where data are collected using the rotation method, as is standard in X-ray crystallography, data-processing software such as *MOSFLM* and *XDS* can be employed to successfully integrate the Bragg peaks. Here, we show that the *DIALS* package, with appropriate adaptations, is also a viable alternative, even for difficult data sets with problematic features such as distortions caused by microscope lens systems and drift of the direct beam position. A set of seven example data sets was successfully processed using *DIALS*, and the specific decisions required at each step are described in detail. The quality of data integrated with *DIALS* is very similar to what could be achieved with *XDS* (Clabbers *et al.*, 2017 ▸).

A major focus of the *DIALS* software is the global modelling of an experiment. The experimental geometry is optimized using all available data. Where components such as the crystal or the beam are expected to change during the course of the experiment, these changes are described using smoothly varying parameterizations, avoiding discontinuities in the model and stabilizing the refinement procedure. Other aspects of interest in global experiment modelling include unit-cell restraints and refinement diagnostics, which enable an exploration of the effects of different parameterizations on refinement stability. A demonstration using simulated data shows that problematic refinement is caused to a significant level simply by the short wavelength and large effective detector distance of electron diffraction experiments, even before additional factors such as instrumental instabilities are considered.

Besides errors occurring from instrumentation, there are additional issues specific to electron crystallography that will need to be addressed. The measured kinematic signal in ED is obscured by inelastic, dynamic and mixed multiple scattering events (Dorset, 1995 ▸; Zou *et al.*, 2011 ▸; Clabbers & Abrahams, 2018 ▸). Zero-loss energy filtering is an instrumental solution to this problem that can filter out most of the inelastically scattered electrons, reducing the diffuse background and sharpening Bragg peaks (Yonekura *et al.*, 2002 ▸). This should improve the accuracy of the intensity estimations from the recorded three-dimensional spot profiles. However, it is not possible to discriminate between kinematic and dynamic scattering energetically. On average, dynamic scattering increases the intensity of weak spots, which become stronger, and the stronger spots become weaker (Weirich *et al.*, 2000 ▸). This directly affects the measured intensities, which form the basis for any further structure determination. These adverse effects are currently not taken into account during data integration.

Electron diffraction of macromolecular crystals is still developing and is confronting crystallographers with new and sometimes unexpected problems. The extensive diagnostics offered by *DIALS*, in terms of corrgrams and its user-friendly, interactive tools for visual inspection of data and parameters, should help in identifying and solving the new challenges specific to optimally integrating electron diffraction data. The toolkit design philosophy of the software, including an extensible image-format reading system and permissive open-source licencing, lowers the barrier to entry for use and future development by scientists interested in this technique.

## Supplementary Material

Supplementary methods, figure and table.. DOI: 10.1107/S2059798318007726/rr5161sup1.pdf

## Figures and Tables

**Table 1:** Details relevant to the modelling of diffraction geometry are collated here for the seven example data sets

Data set	1	2	3	4	5	6	7
Detector distance (mm)	1890	2055	2055	2055	2055	2055	2055
Distortion correction	No	Yes	Yes	Yes	Yes	Yes	Yes
No. of images	503	263	587	419	422	421	421
Image width (°)	0.076	0.1615	0.0344	0.0481	0.0481	0.0481	0.0481
No. of indexed spots	1624	1239	218	598	634	174	211
Condition number† κ(**J**)	1.1 × 10^4^	9.2 × 10^3^	2.0 × 10^4^	2.7 × 10^4^	2.8 × 10^4^	1.1 × 10^4^	2.3 × 10^4^
Static cell‡ (Å)							
*a*	31.967 (7)	32.127 (4)	31.56 (5)	32.36 (2)	31.841 (11)	31.70 (15)	31.63 (2)
*b*	69.41 (3)	68.59 (2)	65.0 (2)	67.25 (6)	65.81 (3)	65.6 (4)	69.08 (5)
*c*	104.62 (3)	104.875 (17)	106.4 (3)	105.71 (7)	103.2 (3)	106.7 (6)	104.07 (4)
Average varying cell (Å)							
*a*	32.0	32.2	31.5	—	31.8	—	31.7
*b*	68.3	68.5	67.1	—	64.7	—	68.8
*c*	105.1	104.9	104.2	—	103.5	—	104.1
Beam centre							
Panel ID	2	0	0	0	0	0	0
*X* (pixels)	485.4–487.7	420.4–420.9	400.5–400.6	428.0	406.3	405.5–406.0	399.1–399.2
*Y* (pixels)	1.7–2.5	478.8–478.9	475.1–477.0	478.3	479.0	480.0–480.6	490.9–491.6
Final r.m.s.d.							
*X* (pixels)	0.93	0.42	0.94	0.67	0.53	0.65	0.51
*Y* (pixels)	0.83	0.59	0.95	0.85	0.66	0.58	0.63
*Z* (°)	0.06	0.08	0.09	0.11	0.04	0.48	0.04

† The condition-number diagnostic is shown for the final step of static refinement.

‡ Errors as reported by *dials.refine* refer to precision estimated by the least-squares refinement procedure and are not indicative of the accuracy of the unit cell. The unit cell for data set 6 was refined with a restraint to an external target.

**Table 2:** Data-processing and refinement statistics for seven merged data sets Values in parentheses are for the highest resolution shell.

Data processing	
Space group	*P*2_1_2_1_2
Unit-cell parameters	
*a*, *b*, *c* (Å)	104.57, 67.62, 31.87
α, β, γ (°)	90.00, 90.00, 90.00
Resolution† (Å)	56.78–2.10 (2.16–2.10)
*R*_merge_	0.313 (0.460)
*R*_meas_	0.356 (0.574)
*R*_p.i.m._	0.160 (0.337)
No. of observations	31650 (1504)
Completeness (%)	59.2 (51.5)
Multiplicity	3.9 (2.7)
〈*I*/σ(*I*)〉	3.0 (1.9)
CC_1/2_ (%)	90.9 (62.6)
Refinement	
Space group	*P*2_1_2_1_2
Unit-cell parameters‡	
*a*, *b*, *c* (Å)	104.45, 67.54, 31.84
α, β, γ (°)	90.00, 90.00, 90.00
Resolution (Å)	56.72–2.10
No. of reflections	8143
*R*1§ (%)	25.2
*R*_complete_ ¶ (%)	29.2
〈*B*〉 (Å^2^)	18.3
R.m.s.*Z*, bond lengths	0.48
R.m.s.*Z*, bond angles	0.72
Ramachandran (favoured/allowed/outliers) (%)	97.6/2.0/0.4

† Individual data sets 1–7 were truncated at CC_1/2_ ≥ 50% and 〈*I*/σ(*I*)〉 ≥ 1.0 (Diederichs & Karplus, 2013 ▸); the merged data set was limited to 2.1 Å resolution based on the model-refinement results.

‡ Unit-cell dimensions after lattice-parameter refinement in *REFMAC*5 (Murshudov *et al.*, 2011 ▸).

§ *R*1 = 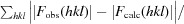 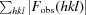, where the sum includes all data.

¶ *R*_complete_ is a robust validation method, especially in cases where the data completeness is limited, making use of all reflections (Brünger, 1997 ▸), and *R*_work_ is thus equivalent to *R*1. *R*_complete_ was calculated with a 0.2% test set size as described in Luebben & Gruene (2015 ▸) and Clabbers *et al.* (2017 ▸).

## Footnotes

1 As the diffraction angle θ→0, arctan(θ) ≃ arcsin(θ) ≃ θ. Substituting this into Bragg’s law and the geometry of the diffraction (where *D* is the detector distance and *r* is the distance between the central beam position and a Bragg spot with resolution *d*) results in *D*/*r* = *d*/λ; with *r* and λ known, *d* and *D* are linearly correlated.

## Appendix A Examples

The following options were written to a file which was passed to *dials.import* in order to override the initial model for experimental geometry of the example data set 1:

Unit-cell restraints to an external target cell were created for data set 6 of the example set by writing the following lines to a file and passing this in at the *dials.index* and *dials.refine* steps:

The correct format for these PHIL files may be explored interactively using command-line switches for *DIALS* programs. For example, the command dials.import -c -a2 -e2 will show all configuration options for this program along with types and help strings up to an ‘expert level’ of 2.

## References

[bb1] Arndt, U. W. & Wonacott, A. J. (1977). *The Rotation Method in Crystallography.* Amsterdam: North-Holland.

[bb2] Bricogne, G. (1986*a*). *Proceedings of the EEC Cooperative Workshop on Position-Sensitive Detector Software (Phases I and II)*. Paris: LURE.

[bb3] Bricogne, G. (1986*b*). *Proceedings of the EEC Cooperative Workshop on Position-Sensitive Detector Software (Phase III)*. Paris: LURE.

[bb4] Brünger, A. T. (1997). *Methods Enzymol.* **277**, 366–396. 10.1016/s0076-6879(97)77021-6 18488318

[bb5] Capitani, G. C., Oleynikov, P., Hovmöller, S. & Mellini, M. (2006). *Ultramicroscopy*, **106**, 66–74. 10.1016/j.ultramic.2005.06.003 16046067

[bb6] Clabbers, M. T. B. & Abrahams, J. P. (2018). *Crystallogr. Rev.*, https://doi.org/10.1080/0889311X.2018.1446427.

[bb7] Clabbers, M. T. B., van Genderen, E., Wan, W., Wiegers, E. L., Gruene, T. & Abrahams, J. P. (2017). *Acta Cryst.* D**73**, 738–748. 10.1107/S2059798317010348 PMC5586247 28876237

[bb8] Dauter, Z. & Wlodawer, A. (2015). *Acta Cryst.* D**71**, 2217–2226. 10.1107/S1399004715015503 PMC4631477 26527139

[bb9] Diederichs, K. & Karplus, P. A. (2013). *Acta Cryst.* D**69**, 1215–1222. 10.1107/S0907444913001121 PMC3689524 23793147

[bb10] Dorset, D. L. (1976). *J. Appl. Cryst.* **9**, 142–144.

[bb11] Dorset, D. L. (1995). *Structural Electron Crystallography.* New York: Plenum.

[bb12] Evans, P. (2006). *Acta Cryst.* D**62**, 72–82. 10.1107/S0907444905036693 16369096

[bb13] Evans, P. R. & Murshudov, G. N. (2013). *Acta Cryst.* D**69**, 1204–1214. 10.1107/S0907444913000061 PMC3689523 23793146

[bb14] Gemmi, M., La Placa, M. G. I., Galanis, A. S., Rauch, E. F. & Nicolopoulos, S. (2015). *J. Appl. Cryst.* **48**, 718–727.

[bb15] Genderen, E. van, Clabbers, M. T. B., Das, P. P., Stewart, A., Nederlof, I., Barentsen, K. C., Portillo, Q., Pannu, N. S., Nicolopoulos, S., Gruene, T. & Abrahams, J. P. (2016). *Acta Cryst.* A**72**, 236–242. 10.1107/S2053273315022500 PMC4770873 26919375

[bb16] Gildea, R. J., Waterman, D. G., Parkhurst, J. M., Axford, D., Sutton, G., Stuart, D. I., Sauter, N. K., Evans, G. & Winter, G. (2014). *Acta Cryst.* D**70**, 2652–2666. 10.1107/S1399004714017039 PMC4188007 25286849

[bb17] Glaeser, R. M., Downing, K. & DeRosier, D. (2007). *Electron Crystallography of Biological Macromolecules.* Oxford University Press.

[bb18] Grosse-Kunstleve, R. W., Sauter, N. K., Moriarty, N. W. & Adams, P. D. (2002). *J. Appl. Cryst.* **35**, 126–136.

[bb19] Gruene, T., Li, T., van Genderen, E., Pinar, A. B. & van Bokhoven, J. A. (2018). *Chem. Eur. J.* **24**, 2384–2388. 10.1002/chem.201704213 29193398

[bb20] Hattne, J., Reyes, F. E., Nannenga, B. L., Shi, D., de la Cruz, M. J., Leslie, A. G. W. & Gonen, T. (2015). *Acta Cryst.* A**71**, 353–360. 10.1107/S2053273315010669 PMC4487423 26131894

[bb80] Hovmöller, S. (2017). 3D Electron Crystallography for Macromolecular Compounds workshop, Paul Scherrer Institute, Switzerland.

[bb35] Joosten, R. P., Long, F., Murshudov, G. N. & Perrakis, A. (2014). *IUCrJ*, **1**, 213–220. 10.1107/S2052252514009324 PMC4107921 25075342

[bb36] Kabsch, W. (2010). *Acta Cryst.* D**66**, 125–132. 10.1107/S0907444909047337 PMC2815665 20124692

[bb37] Keegan, R., Waterman, D. G., Hopper, D. J., Coates, L., Taylor, G., Guo, J., Coker, A. R., Erskine, P. T., Wood, S. P. & Cooper, J. B. (2016). *Acta Cryst.* D**72**, 933–943. 10.1107/S2059798316010433 27487824

[bb38] Khasnis, M. D., Halkidis, K., Bhardwaj, A. & Root, M. J. (2016). *PLoS Pathog.* **12**, e1006098. 10.1371/journal.ppat.1006098 PMC5222517 27992602

[bb39] Kolb, U., Gorelik, T., Kübel, C., Otten, M. T. & Hubert, D. (2007). *Ultramicroscopy*, **107**, 507–513. 10.1016/j.ultramic.2006.10.007 17234347

[bb40] Kolb, U., Gorelik, T. & Mugnaioli, E. (2009). *MRS Proc.* **1184**, 1184-GG01-05.

[bb81] Le Page, Y. (1982). *J. Appl. Cryst.* **15**, 255–259.

[bb41] Leslie, A. G. W. (1999). *Acta Cryst.* D**55**, 1696–1702. 10.1107/s090744499900846x 10531519

[bb42] Leslie, A. G. W. (2006). *Acta Cryst.* D**62**, 48–57. 10.1107/S0907444905039107 16369093

[bb43] Leslie, A. G. W. & Powell, H. R. (2007). *Evolving Methods for Macromolecular Crystallography*, edited by R. Read & J. Sussman, pp. 41–51. Dordrecht: Springer.

[bb44] Luebben, J. & Gruene, T. (2015). *Proc. Natl Acad. Sci. USA*, **112**, 8999–9003. 10.1073/pnas.1502136112 PMC4517205 26150515

[bb45] McCoy, A. J., Grosse-Kunstleve, R. W., Adams, P. D., Winn, M. D., Storoni, L. C. & Read, R. J. (2007). *J. Appl. Cryst.* **40**, 658–674. 10.1107/S0021889807021206 PMC2483472 19461840

[bb46] Mugnaioli, E. & Kolb, U. (2014). *Microporous Mesoporous Mater.* **189**, 107–114.

[bb47] Murshudov, G. N., Skubák, P., Lebedev, A. A., Pannu, N. S., Steiner, R. A., Nicholls, R. A., Winn, M. D., Long, F. & Vagin, A. A. (2011). *Acta Cryst.* D**67**, 355–367. 10.1107/S0907444911001314 PMC3069751 21460454

[bb48] Nederlof, I., van Genderen, E., Li, Y.-W. & Abrahams, J. P. (2013). *Acta Cryst.* D**69**, 1223–1230. 10.1107/S0907444913009700 PMC3689525 23793148

[bb49] Palatinus, L., Brázda, P., Boullay, P., Perez, O., Klementová, M., Petit, S., Eigner, V., Zaarour, M. & Mintova, S. (2017). *Science*, **355**, 166–169. 10.1126/science.aak9652 28082587

[bb50] Parkhurst, J. M., Brewster, A. S., Fuentes-Montero, L., Waterman, D. G., Hattne, J., Ashton, A. W., Echols, N., Evans, G., Sauter, N. K. & Winter, G. (2014). *J. Appl. Cryst.* **47**, 1459–1465. 10.1107/S1600576714011996 PMC4119952 25242914

[bb51] Parkhurst, J. M., Thorn, A., Vollmar, M., Winter, G., Waterman, D. G., Fuentes-Montero, L., Gildea, R. J., Murshudov, G. N. & Evans, G. (2017). *IUCrJ*, **4**, 626–638. 10.1107/S2052252517010259 PMC5619854 28989718

[bb52] Parkhurst, J. M., Winter, G., Waterman, D. G., Fuentes-Montero, L., Gildea, R. J., Murshudov, G. N. & Evans, G. (2016). *J. Appl. Cryst.* **49**, 1912–1921. 10.1107/S1600576716013595 PMC5139990 27980508

[bb53] Pflugrath, J. W. (1999). *Acta Cryst.* D**55**, 1718–1725. 10.1107/s090744499900935x 10531521

[bb54] Read, R. J. & McCoy, A. J. (2016). *Acta Cryst.* D**72**, 375–387. 10.1107/S2059798315013236 PMC4784668 26960124

[bb55] Reeke, G. N. (1984). *J. Appl. Cryst.* **17**, 238–243.

[bb57] Sauter, N. K., Grosse-Kunstleve, R. W. & Adams, P. D. (2006). *J. Appl. Cryst.* **39**, 158–168.

[bb58] Shi, D., Nannenga, B. L., de la Cruz, M. J., Liu, J., Sawtelle, S., Calero, G., Reyes, F. E., Hattne, J. & Gonen, T. (2016). *Nature Protoc.* **11**, 895–904. 10.1038/nprot.2016.046 PMC5357465 27077331

[bb59] Stanton, M. (1993). *Nucl. Instrum. Methods Phys. Res. A*, **325**, 550–557.

[bb60] Stanton, M., Phillips, W. C., Li, Y. & Kalata, K. (1992). *J. Appl. Cryst.* **25**, 549–558.

[bb61] Unwin, P. N. T. & Henderson, R. (1975). *J. Mol. Biol.* **94**, 425–440. 10.1016/0022-2836(75)90212-0 1236957

[bb62] Vainshtein, B. K. (1964). *Structure Analysis by Electron Diffraction.* Oxford: Pergamon Press.

[bb63] Wan, W., Sun, J., Su, J., Hovmöller, S. & Zou, X. (2013). *J. Appl. Cryst.* **46**, 1863–1873. 10.1107/S0021889813027714 PMC3831301 24282334

[bb64] Waterman, D. & Evans, G. (2010). *J. Appl. Cryst.* **43**, 1356–1371. 10.1107/S0021889810033418 PMC4786054 27006649

[bb65] Waterman, D. G., Winter, G., Gildea, R. J., Parkhurst, J. M., Brewster, A. S., Sauter, N. K. & Evans, G. (2016). *Acta Cryst.* D**72**, 558–575. 10.1107/S2059798316002187 PMC4822564 27050135

[bb66] Weirich, T. E., Ramlau, R., Simon, A., Hovmöller, S. & Zou, X. (1996). *Nature (London)*, **382**, 144–146.

[bb67] Weirich, T. E., Zou, X. D., Ramlau, R., Simon, A., Cascarano, G. L., Giacovazzo, C. & Hovmöller, S. (2000). *Acta Cryst.* A**56**, 29–35. 10.1107/s0108767399009605 10874414

[bb68] Winter, G., Waterman, D. G., Parkhurst, J. M., Brewster, A. S., Gildea, R. J., Gerstel, M., Fuentes-Montero, L., Vollmar, M., Michels-Clark, T., Young, I. D., Sauter, N. K. & Evans, G. (2018). *Acta Cryst.* D**74**, 85–97. 10.1107/S2059798317017235 PMC5947772 29533234

[bb69] Yonekura, K., Kato, K., Ogasawara, M., Tomita, M. & Toyoshima, C. (2015). *Proc. Natl Acad. Sci. USA*, **112**, 3368–3373. 10.1073/pnas.1500724112 PMC4372003 25730881

[bb70] Yonekura, K., Maki-Yonekura, S. & Namba, K. (2002). *Biophys. J.* **82**, 2784–2797. 10.1016/S0006-3495(02)75619-1 PMC1302066 11964264

[bb71] Yun, Y., Zou, X., Hovmöller, S. & Wan, W. (2015). *IUCrJ*, **2**, 267–282. 10.1107/S2052252514028188 PMC4392419 25866663

[bb72] Zhang, D., Oleynikov, P., Hovmöller, S. & Zou, X. (2010). *Z. Kristallogr.* **225**, 94–102.

[bb73] Zou, X., Hovmöller, S. & Oleynikov, P. (2011). *Electron Crystallo­graphy.* Oxford University Press.

