# Supplementary material for: Electron diffraction data processing with DIALS
Source: Acta Crystallogr D Struct Biol. 2018 May 30;74(Pt 6):506–18. doi: 10.1107/S2059798318007726 (PMC6096487; doi:10.1107/S2059798318007726)
Supplement: Supplementary file 1 [file d-74-00506-sup1.pdf]

Electron diffraction data processing with *DIALS*
Supporting information

1 Simulation for comparison of ED versus MX geometry refinement

To generate simulated spot centroid positions, we started with the real electron diffraction example *dataset 1*, consisting of a continuous rotation scan over 503 images with an angular width of 0.076° per image, for a total scan range of 38.2°. We took the model for the indexed experiments and "regularised" the geometry of the beam and detector for the purposes of simulation, without changing the crystal model, which had an orthorhombic unit cell with dimensions $a = 31.97$ Å, $b = 69.41$ Å and $c = 104.62$ Å. To regularise the beam and detector models, we forced the beam direction to be exactly aligned to the $-Z$ direction and reoriented the detector model such that the beam intersected the detector in the centre of its square window, and the detector plane was orthogonal to the beam vector. The detector distance remained at the value of 1890 mm, as previously determined and stored in the CBF headers for the images. The real detector consists of 2×2 Timepix quads with large gaps between the active regions. For simplicity we replaced this model with a single panel covering the total extent of the real detector, with no parallax correction, effectively assuming it consists of a perfectly sensitive plane of zero thickness. The updated electron diffraction geometry was written to a new *dxtbx* experiment list and then altered a second time to produce regularised geometry for an X-ray experiment. This involved changing the wavelength from 0.025 08 Å to 1.0332 Å and the detector model such that the total extent and pixel size was equivalent to a Pilatus 6M detector at a distance of 200 mm from the sample. This model was also written to a *dxtbx* experiment list.

The regularised models were used alongside the indexed spot list from the real data set to simulate observed centroid positions for both versions of the experimental geometry. By using the spot list from a real experiment we ensured a realistic distribution of strong spots versus resolution. To make sure that the differences in refinement runs are caused only by the diffraction geometry and not obscured by different sets of input spots, we selected 1571 reflections that could be predicted by both versions of the diffraction geometry.

Simulated centroid positions were calculated for each version of the geometry by predicting their positions then adding random error. The random errors were drawn from a normal distribution with a standard deviation of 0.25 pixels for the X and Y positions and 0.25 images for the Z position. For real data, the centroid position errors in X, Y and Z are neither independent, nor normally-distributed. However, the purpose of adding displacements to the centroid positions was merely to ensure that refinement would proceed to convergence with realistic final RMSDs. The centroid positions from spot-finding result from a centre-of-gravity calculation, which also provides estimated errors in these positions that are used to set weights in refinement. These errors have a dependence on the found spot intensity. Rather than simulating new error estimates, we kept the original error estimates from spot-finding on the real data set to give a realistic distribution of weights. The centroid X, Y positions and their errors were rescaled to units of millimetres for use in refinement using the pixel sizes of 55 µm for the electron diffraction detector and 172 µm for the X–ray detector.

Figure 1: Corrgrams produced for the final step of geometry refinement for simulated data assuming either typical electron diffraction geometry (left column) or X–ray diffraction geometry (right column). The top row shows correlation between effects of different parameters on the positional residuals $(X - X_o)$, the middle row shows those for the $(Y - Y_o)$ residuals and the lower row contains the corrgrams for the angular residuals $(\phi - \phi_o)$.

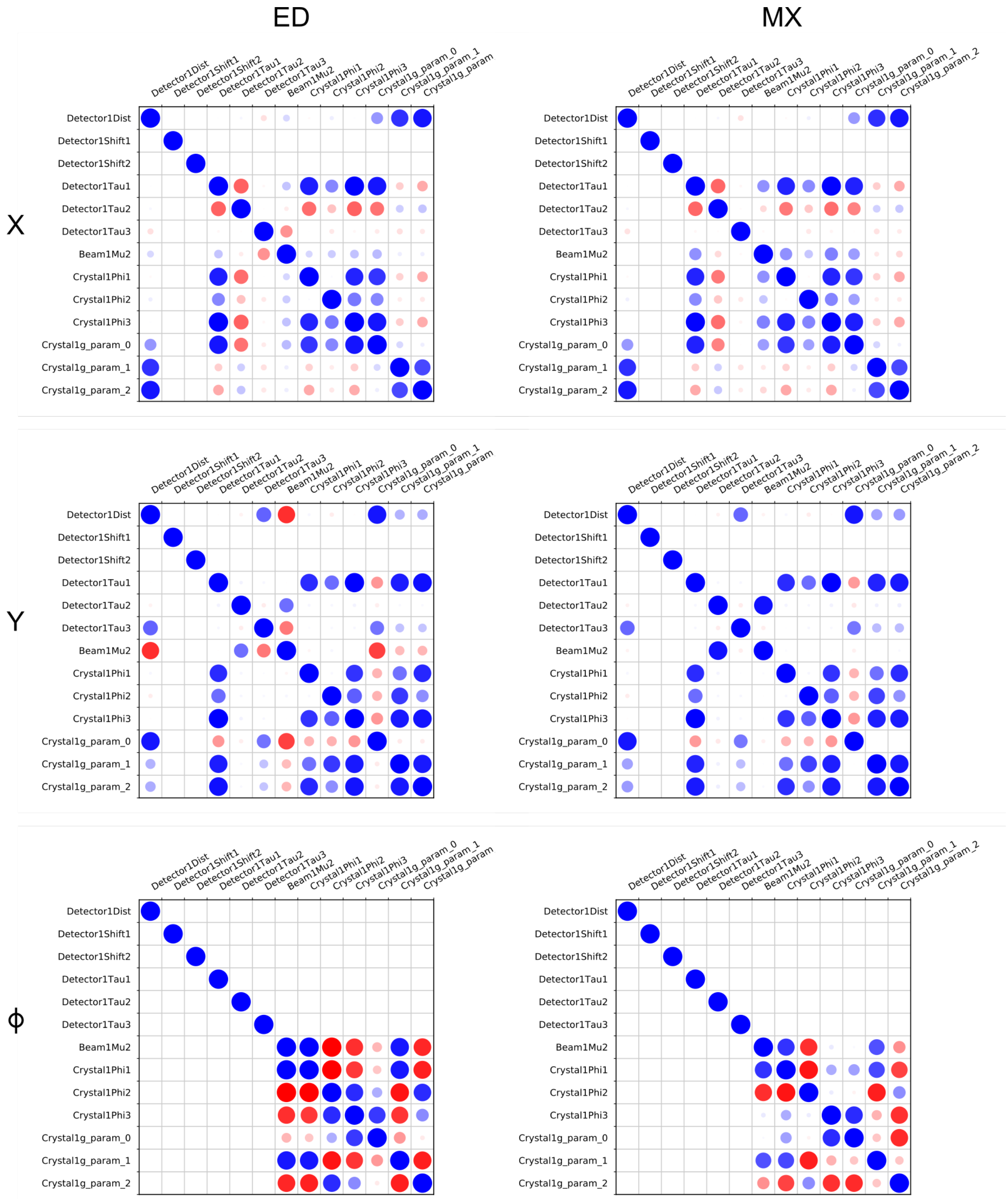

2 Processing statistics for individual datasets

Datasets were processed individually in order to determine suitable resolution cutoffs. These limits were then applied to unscaled data, forming the input to the multiple dataset scaling and merging reported in the main text.

Table S1: Data processing statistics as reported by AIMLESS for 7 individual datasets. Values in parentheses refer to the highest resolution shell.

Dataset	1	2	3	4	5	6	7
Space group	$P2_12_12$	$P2_12_12$	$P2_12_12$	$P2_12_12$	$P2_12_12$	$P2_12_12$	$P2_12_12$
Unit cell							
a (Å)	105.12	104.93	104.25	105.22	103.47	105.00	104.14
b (Å)	68.34	68.51	67.17	69.65	64.73	66.50	68.83
c (Å)	31.98	32.15	31.55	32.35	31.84	31.71	31.73
Resolution (Å)	31.18–2.00 (2.05–2.00)	32.15–2.89 (3.07–2.89)	41.18–2.85 (3.12–2.85)	24.61–2.77 (2.96–2.77)	27.12–2.64 (2.80–2.64)	28.09–3.20 (3.58–3.20)	34.71–3.00 (3.29–3.00)
R_{merge}	0.312 (0.538)	0.218 (0.567)	0.318 (0.538)	0.244 (0.513)	0.248 (0.465)	0.437 (0.613)	0.210 (0.504)
R_{meas}	0.398 (0.667)	0.283 (0.692)	0.437 (0.731)	0.323 (0.659)	0.333 (0.608)	0.583 (0.816)	0.275 (0.665)
R_{pim}	0.244 (0.389)	0.176 (0.381)	0.298 (0.492)	0.210 (0.408)	0.221 (0.387)	0.383 (0.532)	0.175 (0.430)
Observations	18907 (1512)	4983 (946)	2034 (571)	2792 (653)	3141 (601)	914 (276)	2012 (513)
Completeness (%)	50.1 (48.4)	42.0 (41.7)	26.4 (28.6)	26.6 (28.7)	29.7 (30.2)	17.3 (18.1)	25.9 (28.1)
Multiplicity	2.4 (2.8)	2.2 (2.7)	1.5 (1.6)	1.7 (2.0)	1.6 (1.9)	1.4 (1.5)	1.6 (1.6)
$\langle I/\sigma(I)\rangle$	2.0 (1.4)	2.7 (1.5)	2.0 (1.1)	2.2 (1.5)	1.8 (1.4)	2.1 (1.0)	2.2 (1.0)
$CC_{1/2}$ (%)	89.3 (53.1)	91.6 (52.8)	65.5 (51.3)	88.8 (58.8)	85.6 (59.2)	73.1 (55.4)	92.9 (46.4)
